# The relationship between leukocyte to albumin ratio and atrial fibrillation severity

**DOI:** 10.1186/s12872-023-03097-y

**Published:** 2023-02-04

**Authors:** Fabrice Yves Ndjana Lessomo, Qian Fan, Zhi-Quan Wang, Chishimba Mukuka

**Affiliations:** 1 Cardiovascular Internal Medicine, Cardiology Department, Wuhan University Zhongnan Hospital, Wuhan, China; 2 MANSA General Hospital, Mansa, Zambia; 3 Cardiology Department, The second affiliated hospital of Shandong First Medical University, Shandong, China

**Keywords:** Atrial fibrillation, Severity of illness, Leukocyte count, Albumin, CHA2DS2-VASC score

## Abstract

**Background:**

An increased leukocyte count is a sign of inflammation and has been demonstrated to be a predisposing factor and complication of atrial fibrillation. Similarly, albumin, the major protein in the serum, is also considered an acute phase reactant protein that has osmotic and anti-inflammatory properties, and a low albumin level is a known factor associated with severity in many pathologies, including atrial fibrillation. The neutrophil percentage-to-albumin ratio (NPAR) and other emerging leukocyte counts/albumin ratios have been reliable systemic inflammation-based predictors of mortality and complications in various diseases, but they have not yet been used with atrial fibrillation. This study’s aim was to explore whether the leukocyte to albumin ratio could also serve as a useful index in estimating atrial fibrillation severity, including the severity of atrial fibrillation secondary to stroke, to provide a new and more objective tool than the conventional and medical history-based CHA2DS2–VASc score.

**Materials and methods:**

Data were retrospectively collected from the Wuhan University Zhongnan Hospital database from January 1st to December 31st, 2021. The patients were classified into 2 groups: Group 1-low severity and Group 2- moderate to high severity, and diverse statistical analyses were conducted to evaluate the relationship between the leukocyte-to-albumin ratio and AF severity.

**Results:**

Only 2329 test subjects met the inclusion criteria. We had 727 test subjects (381 males and 346 females) categorized into the low severity cohort and 1601 test subjects (932 males and 670 females) in the moderate to high severity group. The difference in mean age between the two groups was significant (95% CI [−2.682 to −0.154] *p* = 0.028), and the difference in the LAR mean rank between the two groups was significant (*p* = 0.00). The Chi-square test of association yielded the following results: the relationship between the LAR level and category of severity was statistically significant (*p* = 0.00), and the Mantel‒Haenszel statistic association odds ratio was OR = 0.657. 95% CI OR [0.549–0.787] *p* = 0.000. The association between sex and atrial fibrillation severity also reached statistical significance. However, sex and LAR were found to be independent factors in atrial fibrillation (Chi-square *p* value = 0.564).

**Conclusion:**

It has been demonstrated throughout this investigation that the leukocyte to albumin ratio could provide key clues in clinical practice and contribute to thromboembolism risk assessment in the setting of atrial fibrillation.

## Background

Atrial fibrillation is an abnormal heart rhythm pattern characterized by the presence of an irregular R-R interval on the ECG, where P waves disappear and are replaced by F waves. AF is a major public health concern, affecting more than 30 million individuals worldwide. Patients with atrial fibrillation are exposed to a higher risk of mortality due to stroke; however, it can be observed that despite recent advances in the identification of parameters linked to atrial fibrillation onset and severity, the recent advances in the ways and means to monitor them, and the establishment of a medical history-based scoring system, the CHA2DS2-VASc score, to predict the risk of stroke in recent decades, the trend of mortality attributed to atrial fibrillation has remained a matter of high concern [1, 2]. This could likely indicate that there is still room for improvement to bring about a considerable decrease in the trend of atrial fibrillation-induced mortality; therefore, it would be of paramount importance to identify all possible indicators or indices pointing to its severity.

Inflammation has been demonstrated to be both a predisposing factor and a complication of atrial fibrillation and vice versa. An increased white blood cell count would indicate inflammation and physiological stress, which means that an increased leukocyte count could be considered a factor indicating the worsening of illness even with underlying atrial fibrillation [3]. Similarly, albumin, the main protein found in the serum, is regarded as an acute phase reactant protein with osmotic and anti-inflammatory properties. The severity of various diseases, including atrial fibrillation, is known to be correlated with low albumin levels [4]. Although the exact mechanisms are still unclear, it is known that leukocyte activation plays a critical role in the prevalence of atrial fibrillation and may contribute to the amplification of structural remodelling and the associated damage. In addition, hypoalbuminemia is said to be a predisposing factor in the risk of oxidation and thromboembolism, which could also potentiate a worse prognosis in atrial fibrillation and stroke.

Recently, many new indices derived from the leukocyte count or percentages and albumin, such as NLAR, LAR, and NPAR, have been proposed as prognostic markers, and those indices have proven to be more sensitive than conventional systemic inflammation markers for predicting mortality or other poor outcomes of coronavirus pneumonia, heart failure, hepatitis, cirrhosis, STEMI, and cardiogenic shock. For instance, the neutrophil percentage-to-albumin ratio (NPAR) has been shown to be a reliable systemic inflammation-based predictor of mortality in a variety of diseases [5, 6]. Thus far, most of the studies that previously assessed the association between atrial fibrillation and systemic or severe inflammation did not include the leukocyte count and albumin-derived ratios.

In clinical practice, efforts and research are deployed to more comprehensively manage the disease. To date, the management of atrial fibrillation has revolved around the CHA2DS2-Vasc score, an updated version of the CHA2DS2 score that is used to estimate the risk of stroke in patients with atrial fibrillation. AF can cause blood stasis in the upper heart chambers, leading to the formation of a mural thrombus, which can dislodge into the blood flow, reach the brain, cut off the blood supply to the brain, and cause a stroke. This score is used to determine whether anticoagulation therapy is needed. A total score greater than 1/10 in males and greater than 2/10 in females constitutes an indication to implement anticoagulation management. However, this scoring system seems to be more subjective than objective, and despite the improvements observed, there is still a need for readily inexpensive and more objective indices. Therefore, the aim of this study is to explore whether the leukocyte to albumin ratio (LAR) could also serve as a marker or an indicator of atrial fibrillation severity, including the severity of atrial fibrillation with stroke, to widen the arsenal of a clinical index so that it can be considered in the management of atrial fibrillation.

## Materials and methods

### Data collection and study population

To achieve the aim of this study, data were retrospectively collected from the electronic clinical registries database of Wuhan University Zhongnan Hospital. With prior approval from the hospital ethical committee, ECG room records of atrial fibrillation from January 1st, 2021 to December 31st, 2021 were gathered, analysed and grouped by hospital department. Patient relevant information, such as sex, age, and lab investigations, including only the following parameters: complete blood count, albumin value in g/L, BNP, PRO BNP, D-dimer, and HSTNI, were retrieved and recorded on an Excel worksheet, were then translated from Chinese to English and then were converted into an SPSS document. In this retrospective cohort study, the patients were classified into 2 groups: Group 1-low severity and Group 2- moderate to high severity. The atrial fibrillation severity was determined by elevated levels of BNP (> 200 pg/ml), PRO BNP (> 2000 pg/ml), HSTNI (> 75 pg/ml) and D-dimer (> 1500 ng/ml).

### Inclusion and exclusion criteria

To be included in this study, patients had to have met the following conditions: patients should have been admitted to Zhongnan Hospital between January 2021 and December 2021, irrespective of the department or branches, with complete blood count, liver function test, BNP or PRO-BNP, HSTNI, D-DIMER reports available. To be included in the moderate to severe group, the patients were to present with one or more parameters (BNP, DDIMER, HSTNIPRO PRO-BNP) with a level higher than the threshold defined above. The patients with parameter values lower than the set up threshold were labelled as having low severity. All patients who did not fulfil the inclusion criteria or whose reports were incomplete or empty were excluded.

### Statistical analyses

LAR continuous data were converted into categorical data. A value of 0.2 was considered a normal value for LAR and was obtained by dividing the normal upper value of leukocyte count (10.10^3^) by the normal upper value of albumin (50 g/l). The individuals having LAR < 0.2 were categorized as having low LAR, and those with LAR > 0.2 were categorized as having raised LAR. We performed descriptive statistics for continuous data when the normality test violated the Mann‒Whitney test for independent samples to compare the distribution of LAR between the two groups. We also performed an independent samples T test to compare the mean age between the low and high LAR clusters. For categorical data, a Chi-square test of the association between categories of LAR and severity of atrial fibrillation and between LAR and sex was performed, and the odds ratios were determined by Mantel‒Haenszel methods. All statistical analyses were performed with IBM SPSS STATISTIC 22 software.

## Results

**Fig. 1:**
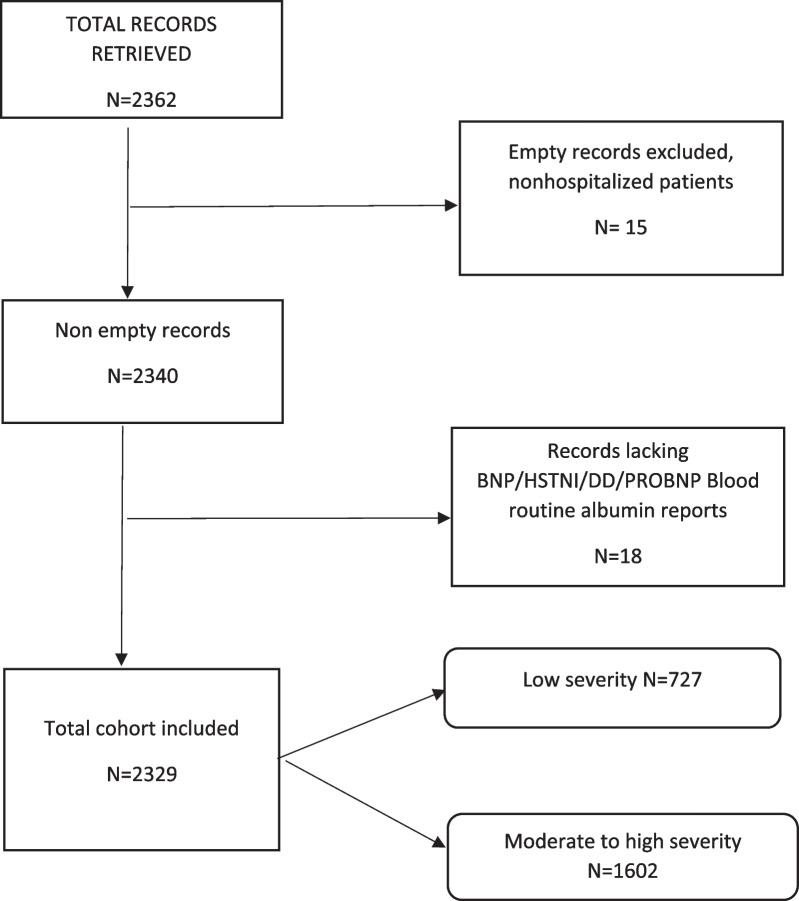
Case selection process

A total of 2362 reports of atrial fibrillation records were retrieved from the hospital registries and from more than 40 different departments accounting for the year 2021 (from January to December 2021). Then, the patients were screened to check for their eligibility for this study, and the screening process and the characteristics of the cohort are illustrated in the charts below (Fig. 1, Table 1).

**Table 1:** Characteristics of the participants

Variables	Low severity n (%)	Moderate to high severity n (%)	*p* value
Participants	727	1602	
Sex (male/female)	381 (52.4)/3469 (47.6)	932 (58.2)/670 (41.8)	0.009
Age (mean)	68.45	69.87	0.028
LAR (mean*10^3^) (median*10^3^)/	0.2096/0.1769	0.2453/0.1944	0.000

This table shows the variations observed across the involved cohorts. The values represented by n indicate the mean or the median for continuous variables and count for nominal data; (%) represents the percentage for categorical variables only; LAR = leukocyte count to albumin ratio

These results showed that there was statistical significance in the LAR mean difference between the low severity and moderate to high severity groups (*p* = 0.000). Therefore, it can be hypothesized that increased LAR in patients with atrial fibrillation could indicate severity. These results showed that the difference in the mean LAR between the low severity and moderate to high severity groups was statistically significant (*p* = 0.000). Therefore, it could be hypothesized that increased LAR in patients with atrial fibrillation could indicate severity.

**Fig. 2:**
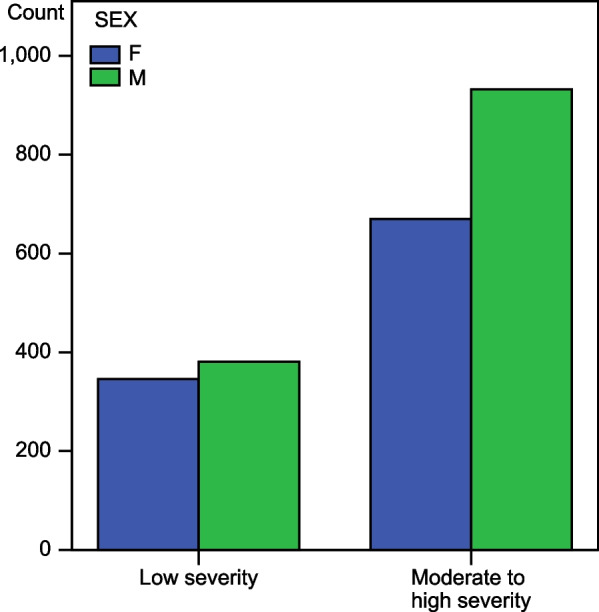
Association between SEX and severity in atrial fibrillation. This bar chart obtained from the Chi-square frequency table analysis on the association between gender and AF severity illustrates the distribution of sex gender (M = male, F = female) among the atrial severity cohorts and the clear predominance of male gender in the high severity group

**Fig. 3:**
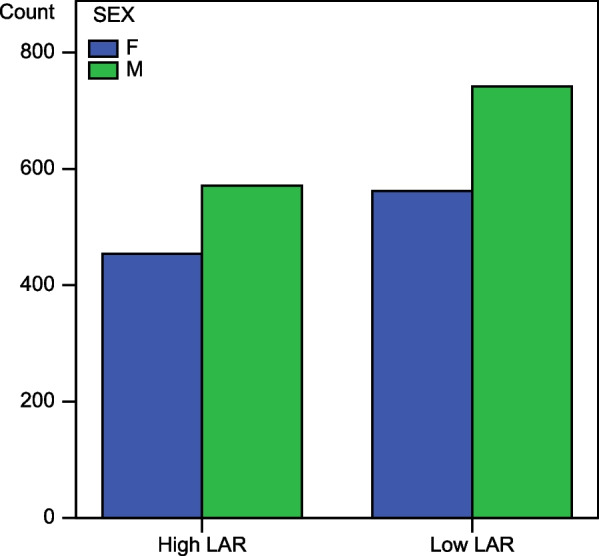
Relationship between LAR and Sex gender in atrial fibrillation. High LAR = cohort with leukocyte to albumin ratio higher than the set threshold; Low LAR = cohort with leukocyte to albumin ratio lower; M = male gender, F = female gender. This chart shows that gender distribution has the same trend in both cohorts with no tangible difference between males and females

We conducted a subgroup analysis to compare the medians of LAR according to the parameter groupings (Table 2), and the results showed that the LAR distribution among the various parameter groupings was not the same. On the independent sample t test, the 95% CI of the mean age difference between the low and high LAR cohorts was [−0.55 to −0.16] *p* = 0.000, showing statistical significance; thus, it could be inferred that with underlying atrial fibrillation, the higher the age was, the higher the LAR. Those obtained from the Chi-square test of independence on the Phi Cramer modality illustrated that the association between LAR level and category of severity was statistically significant (*p* = 0.00), and on the Mantel‒Haenszel statistic, the odds ratio was OR = 0.657. 95% CI OR [0.549–0.787] *p* = 0. 000. The relationship between sex and atrial fibrillation severity also reached statistical significance, and male patients with atrial fibrillation would most likely present with increased atrial severity parameters, as illustrated by the chart in Fig. 2. However, there was no significant association between the LAR categories and sex, so LAR and sex could be considered independent variables in atrial fibrillation (Fig. 3).

**Table 2:** The distribution of the parameters that were increased among the moderate to high severity group

Parameters	Frequency	Percentage	Valid percentage
Not increased	727	31.2	31.2
A	62	2.7	2.7
BNP	183	7.9	7.9
BNP, DD	252	10.8	10.8
DD	379	16.3	16.3
HS	31	1.3	1.3
HS, DD	38	1.6	1.6
HS, PROB	52	2.2	2.2
PROB	265	11.4	11.4
PROB, DD	340	14.6	14.6
Total	2329	100.0	100.0

Data are presented as frequencies and percentages. A = all parameters were increased beyond the threshold, HS = high sensitivity troponin, DD = di-dimer, PROB = Pro BNP

## Discussion

According to the findings of these analyses on the relationship between the leukocyte to albumin ratio and the severity of atrial fibrillation, LAR and increased BNP, DD PROBNP and HSTNI are the variables associated with atrial fibrillation. The distribution of LAR among atrial fibrillation cases varies with the severity profile, and the value of LAR may vary with increasing age but not with sex.

Atrial fibrillation is a condition in which the sinus rhythm is disrupted, causing an irregular pattern of atrioventricular contraction, atrial enlargement with structural remodelling, and a risk of thrombus formation in the left upper chamber of the heart, all of which may contribute to the onset of ischaemic stroke and heart failure. Although the cause of the disturbance is still unclear, there is broad agreement that the pulmonary veins could be the site where the abnormal depolarizing signal originates. According to the current risk score of embolization in atrial fibrillation, anticoagulation therapy would not be recommended or is just considered for some patients. However, clinically among these low-risk populations, some patients were still found to develop thromboembolic events, and further investigation would then be necessary to achieve a better embolization risk classification and optimized anticoagulation therapy assignment in patients with underlying atrial fibrillation. To do this, there is a need to make use of all relevant and reliable scientific indices from parameters that could be associated with embolism and the severity of atrial fibrillation.

In routine clinical practice, leukocyte count has been used to detect signs of inflammation, infection, or even tumours [7]. However, as medical practice evolves, new clinical applications of total or differential leukocyte counts emerge. For example, there is substantial evidence of an association between high mortality in heart failure and increased neutrophil count [8, 9], even if the exact mechanism is still unknown. Albumin, on the other hand, is the most abundant protein in the body; the level of albumin in the serum not only reflects a person's nutritional status but also reflects the liver's synthetic ability. Previous research has thoroughly investigated the clinical and predictive value of albumin in severe diseases, including inflammatory states, and it has been observed that albumin levels seemed to decrease in disease. The distribution of albumin between the intravascular and extravascular compartments would be altered by critical illness. The other changes included changes in the rates of protein synthesis and breakdown. During the early course of serious illness, serum albumin concentrations would frequently decrease sharply and would not increase again until the sickness has subsided [10]. Albumin levels have prognostic value in a variety of diseases, including COPD, stroke, atrial fibrillation, and heart failure. Low albumin levels cause a decrease in oncotic pressure, which causes the development of oedema and, as a result, hypovolemia, a hypoperfusion state (ischaemia), and a decreased glomerular filtration rate. Hypoalbuminemia has also been consistently associated with increased susceptibility to infection and the accumulation of insoluble elements such as iron, copper, bilirubin, etc. All of these are known to be dangerous to human organs, systems and tissue homeostasis. Despite the fact that albumin levels increase slightly in the early stages of inflammatory states, low albumin levels have been found to be associated with inflammation [11–13]. Tissue necrosis is caused by ischaemia, haemodynamic instability, or an abnormal accumulation of metabolites, and the activation and proliferation of immune cells are mediated by the release of chemokines and other mediators, which also secrete toxic substances and free radicals, all of which have the potential to exacerbate the condition and worsen outcomes [14]. These findings would suggest that a low albumin level could trigger both inflammation and an increase in the leukocyte count, whereas severe inflammation can impair liver synthesis and result in hypoalbuminemia. In both cases, the natural progression would be towards a worse outcome.

It has been proven that low albumin is linked to stroke and atrial fibrillation severity. Patients with atrial fibrillation and/or other cardiovascular diseases were shown to have an increased risk of cardio-thromboembolic events when their serum albumin levels were low. Low albumin levels are a strong predictor of atrial fibrillation following coronary artery bypass graft surgery in myocardial infarction, and prior research has shown that stroke patients with poor outcomes in the general population also had considerable hypoalbuminemia. On the other hand, those who had high levels of albumin had better outcomes [15–17]. Several ideas could account for the relationship between atrial fibrillation severity and an elevated leukocyte count. Severe atrial fibrillation is almost if not always associated with left atrial enlargement, which could suggest that during atrial fibrillation episodes, the atrial wall undergoes some electrical, structural, and fibrotic remodelling that may involve some inflammatory response, increasing the recruitment and activation of white blood cells. Maixent et al. showed that circulating myosin-specific autoantibody was present in a significant portion of paroxysmal atrial fibrillation patients, raising the possibility of an ongoing autoimmune process in those patients. If there is autoimmunity, then a raised leukocyte count would be the logical observation. A genetic study discovered that patients with atrial fibrillation and stroke showed some changes with a strong tendency for increases in blood cell expression and activation profiles, and this was attributed to 43 genes specific to AF [18, 19].

As has been demonstrated for heart failure and other diseases, it therefore seems appropriate to consider those two factors when assessing whether a condition is growing severe or may indicate an adverse evolution. Additionally, a multiparameter index's impact would be superior to a single parameter index in terms of performance, sensitivity, and predictive value. The results of our analyses (Tables 2, 3, 4) are also very consistent with all the above observations, plus the statistical significance in the difference in the mean age between the low severity cohort and the moderate to high severity cohort, with the latter being higher (95% CI [−2.682 to −0.154] *p* = 0.028.), was an expected finding showing that this grouping pattern did not deviate from the standard because it is known that the older a person is, the higher their risk of thrombo-embolic events. The theory being investigated here, which holds that the leukocyte to albumin ratio (LAR) could also stand as another new index of disease severity, could be supported by the significant association (*p* < 0.05) between the increase in the severity parameters and the level of LAR on the Chi-square test, which added to the statistical significance in the LAR distribution across the categories of severity on the Mann‒Whitney independent samples test. In addition, age and LAR were found to be dependent variables in atrial fibrillation (*p* < 0.05) (Table 4). Therefore, if age is a key parameter in determining the risk of atrial fibrillation severity in current practice, LAR could also apply. LAR and sex were found to be independent variables in AF, which would imply that LAR levels did not vary according to gender (see Fig. 3), so the new index would be applicable to all genders.

**Table 3:** The distribution of variables observed among the two LAR subgroups and the trend of association with LAR

	Low LAR Cohort n (%)	High LAR Cohort n (%)	*p* value
AF severity {Moderate to High Severity/Low Severity}	846 (64.9)/458 (35.1)	756 (73.8)/269 (26.2)	0.000
Sex: (Male/Female)	571 (56.9)/562 (43.1)	742 (55.7)/454 (44.3)	> 0.05
Age (mean)	68.97	70.01	0.000

The values are represented by n = count or mean (%) and represent the percentage for categorical variables only. LAR = leukocyte to albumin ratio

**Table 4:** Statistical analysis summaries

	Statistics	p value Two tailed	Size effects and 95% CI	Inference
Association between LAR and severity	Pearson Chi-square + Mantel Haenszel	*p* = 0.000 Statistically significant	OR = 0.657 95% CI OR [0.549–0.787]	LAR and increased BNP,DD PROBNP OR HSTNI are the dependent variables in AF
Association between Sex and Severity	Pearson Chi-square + Mantel Haenszel	*p* = 0.009 Statistically significant	OR = 0.14 95% CI OR [−0.032 to 0.321]	Sex and severity are the dependent variables in AF
Association between LAR and sex	Pearson Chi-square + Mantel Haenszel	*p* = 0.564 Not significant	OR = 1.050 95% CI OR [0.890–1.238]	Sex and LAR are the independent variables in AF
Comparison of LAR distribution between the severity categories	Independent sample Mann‒Whitney U test	*p* = 0. 000 Statistically significant	Mean rank difference	The distribution of LAR is not the same across category of severity
Comparison of mean age according to severity	Independent sample t test	*p* = 0.028. Statistically significant	95% CI Mean difference [−2.682 to −0.154]	Advanced age is a factor for severity in AF
Comparison of mean age according to LAR clusters	Independent sample t test	*p* = 0.000 Statistically significant	95% CI Mean difference [−0.55 to −0.16]	LAR levels have increased with age

This table presents the summary of all the statistical analyses performed and the interpretations of their results. LAR = leukocyte to albumin ratio

The significance of these findings resides in the fact that the current clinical practice guidelines advocate more on the CHA2DS2-VASc and HAS-BLED scores to evaluate the severity of atrial fibrillation. Although these tools are very effective, they appear limited, as they rely more on patient history [20, 21] and are thus more subjective than objective. Adding or including a more objective tool or index such as the LAR to the above may certainly bring more effectiveness in the timely identification of high-risk patients and the early management and prevention of terrible outcomes. As the thromboembolism risk remains the main problem in atrial fibrillation, this index could also provide some clues about the thromboembolism risk. Because previous studies asserted that there was a modest association between low serum albumin and thromboembolism events in a larger cohort, which makes low serum albumin levels a clinical marker for thromboembolism [21, 22], a higher leukocyte count has been significantly associated with thromboembolism [23, 24]. Although the exact mechanism of these associations remains unclear, taking into account these reports, it would appear that the LAR would be an index or tool that could be useful in estimating the thromboembolism profile in patients presenting with evidence of atrial fibrillation, thus complementing the conventional CHA2DS2-Vasc score. Moreover, the leukocyte count-to-albumin ratio, such as the CHA2DS2-VASc score, would be applicable to both males and females because there would be no sex discrimination, making this index suitable for clinical use.

However, in this study, some limitations could be noted, and the retrospective nature of this investigation could have given rise to some biases. First, the number of exposed (moderate to high severity group) did not match the number of nonexposed (low severity group), and this could be explained by the fact that for a better validity of the results, it was preferable that the exposed and nonexposed patients be sampled from the same place and in the same interval of time. Our set interval of time was the entire year 2021, but unfortunately, the number of patients who did not present with raised BNP, PRO BNP, DIDIMER OR HSTNI was relatively smaller. Second, the measure of severity only took into consideration the value of BNP, PRO BNP, DIDIMER, and HSTNI; because of the retrospective nature, it is possible that confounding factors exist and might have had an influence on the results obtained. Nevertheless, the significance of the associations and dependence relationship obtained remain worthy of consideration; moreover, to date, this is the first investigation to find that the leukocyte to albumin ratios could provide some clues for atrial fibrillation patients and can be applied in routine clinical practice for an optimum, effective and more objective management of those patients.

## Conclusion

Atrial fibrillation remains a hot topic in cardiovascular medicine, and the increased levels of BNP, DDIMER, PRO BNP, and HSTNI are the factors that correlated with atrial fibrillation severity [25–28] in this study (Table 2). Low albumin and increased leukocyte counts are also known to be very important parameters of atrial fibrillation severity. The leukocyte to albumin ratio could provide key clues in clinical practice and in thromboembolism risk assessment in the setting of atrial fibrillation. However, because the outcome of severity was judged by the parameter value rather than the occurrence of real events, at times patients could indeed present with a very high value of a certain parameter because of other factors. Therefore, more elaborate prospective, retrospective, clinical trial, and survival analysis studies are needed to strengthen the findings reported in this paper. Meanwhile, it would be good to estimate the LAR index in addition to the CHA2DS2-VASc score, in every atrial fibrillation patient; this could bring some net benefits for anticoagulation recommendations, especially in those with low CHA2DS2-VASc scores but who present with increased LAR.

## Data Availability

The datasets used and/or analysed during the current study are available from the corresponding author on reasonable request.

## Abbreviations

AF: Atrial fibrilation
LAR: Leukocyte to albumin ratio
BNP: Brain natriuretic peptide
PROBNP: BNP precursor
DD: Di-dimers
HSTNI: High sensitive troponin I
NPAR: Neutrophil percentage to albumin ratio
NLAR: Neutrophil lymphocyte ratio to albumine ratio
STEMI: ST segment elevated myocardial infarction
COPD: Chronic obstructive pulmonary disease
OR: Odds ratio
CHA2DS2-VASc SCORE: A scoring used to assess the risk of stroke and is comprised of 8 items: Congestive heart failure(score1), Hypertension(score1), Age > 75 (scores 2), Diabetes mellitus(score1), Stroke or TIA (scores 2), Vascular disease(score1), age (64–74)(score1), female Sex Category(score1)
HAS-BLED SCORE: A scoring used to assess the risk of bleeding; it includes hypertension, abnormal liver and/or renal function, stroke, bleeding history, labile INR, elderly (age > 65), and drug and/or alcohol use
